# Effects of methylphenidate on quantitative measures of motor function: a systematic review

**DOI:** 10.3389/fnhum.2026.1756629

**Published:** 2026-04-01

**Authors:** K. Riis, A. R. Brittain, A. DadeMatthews, C. L. Watson, A. B. Grabowsky, K. A. Neely

**Affiliations:** 1 School of Kinesiology, Auburn University, Auburn, AL, United States; 2 Research Support Department, Auburn University Libraries, Auburn University, Auburn, AL, United States

**Keywords:** attention-deficit/hyperactivity disorder (ADHD), fine motor skills, gross motor skills, methylphenidate, motor control, motor performance, systematic review

## Abstract

**Introduction:**

Attention-deficit/hyperactivity disorder (ADHD) is a common mental disorder characterized by persistent patterns of inattention, hyperactivity, and impulsivity. Methylphenidate (MPH) is widely used to manage ADHD symptoms and improve cognitive and behavioral functioning; however, its effects on motor performance remain poorly understood. Most research has focused on cognitive and behavioral outcomes, with limited attention to objective measures of movement.

**Objective:**

This systematic review examined human studies that quantitatively assessed the influence of MPH on motor tasks and movement-related outcomes.

**Methods:**

Following PRISMA guidelines, a comprehensive search utilizing key terms was conducted using four databases: PubMed, APA PsycINFO, International Pharmaceutical Abstracts, and ProQuest Dissertations and Theses Global. Eligible studies were in English, included all age groups, included quantitative data on motor output, and employed observational, cohort, or cross-sectional designs. Additionally, studies must have compared MPH conditions to conditions without MPH. Covidence facilitated two-phase screening, and study quality was appraised using the Quality Assessment Tool for Quantitative Studies.

**Results:**

Of 1,314 titles and abstracts screened, 329 articles underwent full-text review, and 13 met inclusion criteria. Despite considerable methodological heterogeneity and variability of motor tasks, the overall evidence suggests that methylphenidate was associated with improvements in some quantitative motor outcomes, particularly measures of motor variability and steadiness. However, the effects were inconsistent across tasks and mean performance measures often showed no change.

**Conclusion:**

These findings highlight the need for future research to adopt standardized task protocols and consistent outcome measures, enabling more robust comparisons across studies and supporting meta-analytic synthesis.

## Introduction

Attention-deficit/hyperactivity disorder (ADHD) is a common childhood-onset mental disorder characterized by chronic inattention and/or hyperactivity-impulsivity that leads to functional impairments in multiple domains (American Psychiatric Association, 2013). The overall international prevalence of ADHD in children and adolescents is 8% (Ayano et al., 2023), and the prevalence of symptomatic adult ADHD is 6.67% (Song et al., 2021). ADHD may be the result of structural and functional impairments in specific brain regions (Konrad and Eickhoff, 2010; Schneider et al., 2010), particularly those regions involving dopamine and norepinephrine in the prefrontal cortex, basal ganglia, and locus coeruleus (Biederman, 2005). Further, in addition to proposed regional brain abnormalities, some models suggest dysfunction in distributed network organization (Sergeant et al., 2003; Konrad and Eickhoff, 2010).

Methylphenidate (MPH) is a central nervous system stimulant frequently and effectively used as a first-line pharmacologic treatment for ADHD (Kutcher et al., 2004; Rostain and Ramsay, 2006; Greenhill et al., 2002). MPH increases the activity of the central nervous system through inhibition of reuptake of the neurotransmitters norepinephrine and dopamine (Butcher et al., 1991; Leonard et al., 2004), leading to an increase in extracellular dopamine in the brain, particularly in the basal ganglia (Volkow et al., 2002; Volkow et al., 1994; Castellanos et al., 1996). MPH is effective in treating behavioral symptoms of ADHD because it enhances signal processing in the prefrontal cortex (Devilbiss and Berridge, 2008; Salek et al., 2012) and nucleus accumbens (Chong et al., 2012; Claussen et al., 2014), and attenuates activity in the reticular formation and thalamus (Shih et al., 1975). Magnetic resonance imaging (MRI) studies suggest that long-term treatment with MPH may decrease abnormalities in functional activity for individuals with ADHD (Hart et al., 2013).

The literature examining the effect of MPH on movement and motor tasks is equivocal (Kaiser et al., 2015). Previous work evaluating the effects of MPH on movement-related variables include studies of fine and gross motor skills (Kaiser et al., 2015; Polotskaia, 2008; Gualtieri et al., 1986), driving (Cox et al., 2000; Barkley et al., 2005), handwriting (Tucha and Lange, 2004; Lufi and Gai, 2008), exercise (Swart et al., 2009; Medina et al., 2010), play (Barkley and Cunningham, 1979), and activity (Cunningham et al., 1985). Importantly, despite these studies on movement, there has been limited emphasis on objective, quantitative measures of motor output. The lack of a standardized approach to assess the effects of MPH on motor output makes a meta-analytic approach difficult. Therefore, this systematic review aims to evaluate the impact of MPH on quantitatively measured motor outcomes in human studies.

## Methods

### Search strategy

This systematic review focused on the effects of MPH on objective measurements of movement. Reporting of this systematic review follows guidelines established by the PRISMA (Preferred Reporting Items for Systematic Reviews and Meta-Analyses) statement (Page et al., 2021). Search strategies were developed by a health sciences librarian (ABG) with experience in evidence synthesis and included subject headings, synonyms, and related terms for the concepts of MPH and motor skills, activities, or tasks. The searches were conducted in four databases: PubMed, APA PsycINFO (via the EBSCO platform), International Pharmaceutical Abstracts (via the Ovid platform), and ProQuest Dissertations and Theses Global. Initial searches were run on 9/30/2019 with no date limit. Follow-up searches were run on 2/19/2025 and limited to 9/1/2019 to 2/19/2025. All searches were also limited to the English language. Complete search strategies for each database are included in the Appendix.

### Selection process

**Figure 1:**
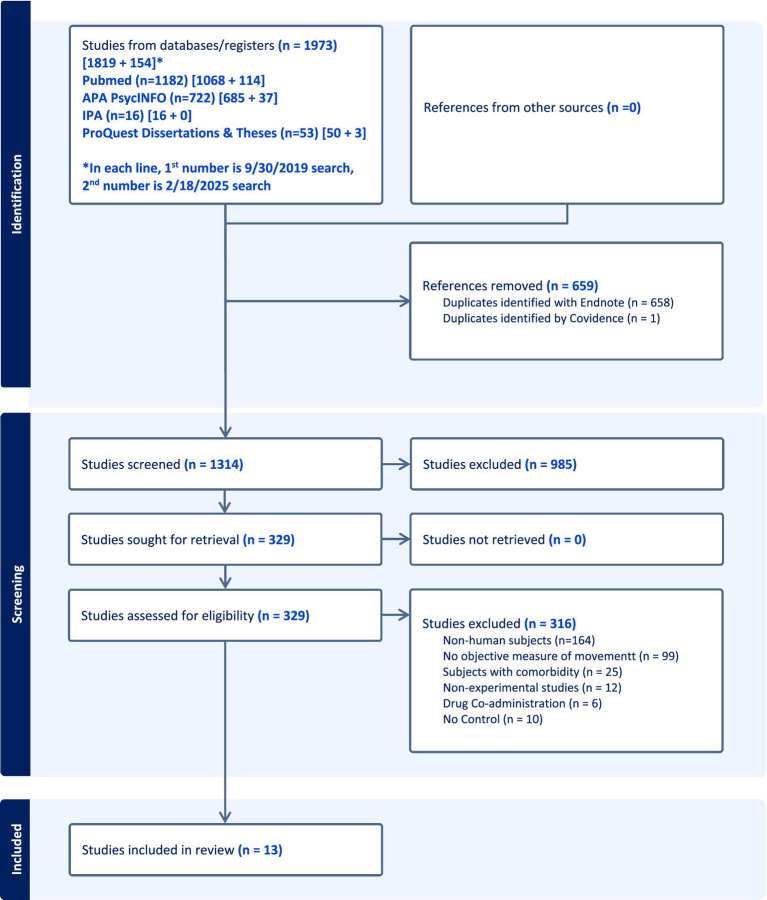
PRISMA flow diagram detailing the study selection process for the systematic review.

Screening was conducted at two levels: title/abstract and full text. For the initial searches, screening was completed using Excel spreadsheets, while Covidence screening software^1^ was used for screening the results of the follow-up searches. As shown in Figure 1, the total of both searches yielded 1,973 results, with 1,314 remaining after duplicates were removed. Titles and abstracts of the remaining 1,314 articles were screened according to the following inclusion criteria: studies had to be primary research articles reporting original data, including observational, cohort, or cross-sectional designs. Studies were required to include and report results of at least one quantifiable measure of purposeful motor output, explicitly focusing on functional motor tasks such as finger tapping, handwriting, gait, balance, or manual tracking. Additionally, studies must have compared MPH conditions to a condition without MPH. Exclusion criteria included non-human studies and secondary research, such as books, reviews, systematic reviews, meta-analyses, editorials, commentaries, letters, meeting abstracts, case studies, and laboratory notes. Studies were also excluded if they involved the simultaneous administration of more than one pharmaceutical treatment with MPH or if participants presented with comorbid conditions. While excluding studies with comorbid conditions may limit generalizability, this approach was necessary to establish a baseline of evidence on methylphenidate administration in ADHD.

Reviewers (ADM, ARB, KR, and CW) independently screened titles and abstracts for eligibility. Of the 1,314 titles and abstracts reviewed, 985 failed to meet the inclusion or exclusion criteria. Conflicts were resolved by a third reviewer (KAN), and a consensus discussion among all reviewers yielded 329 articles for full-text review. Common exclusion reasons during this stage entailed: non-human subjects, non-experimental studies, and did not include MPH. Full texts of the remaining 329 articles were assessed for eligibility, using the same inclusion and exclusion criteria as described above. Reviewers (ADM, ARB, KR, CW, KAN) independently screened full texts for eligibility. As before, conflicts were resolved by a third reviewer and consensus discussion among all reviewers. Thirteen articles remained after full text screening and are included in this review.

### Quality assessment/risk of bias

The quality of each study was assessed using the Quality Assessment Tool for Quantitative Studies (Thomas et al., 2004). This tool assesses the risk of bias and quality of quantitative studies. Two reviewers rated each article independently, and a third reviewer resolved any conflicts. The final scores reported were based on a consensus discussion among all three reviewers. Final scores are reported in Table 1. The tool covers six domains, including selection bias, study design, confounders, blinding, data collection methods, and withdrawals/dropouts. Subsequently, a global rating score is derived based on the individual domain scores. Additionally, the quality of evidence for each study included was evaluated by examining the levels of evidence using Sackett’s levels of evidence (Sackett, 1989):

Level I: Systematic reviews, meta-analyses, randomized controlled trials (RCT).
Level II: Two groups, nonrandomized studies (e.g., cohort, case–control).
Level III: One group, nonrandomized (e.g., before and after, pretest and post-test).
Level IV: Descriptive studies that include analysis of outcomes (single-subject design, case series).
Level V: Case reports and expert opinions that include narrative literature reviews and consensus statements.

**Table 1:** Quality assessment table.

Reference	Selection bias	Study design	Confounders	Blinding	Data collection methods	Withdrawals and dropouts	Global rating
Aitken et al. (2024)	3	1	1	1	1	2	2
Barkley (1977)	3	1	1	2	3	3	3
Barkley et al. (2005)	2	1	1	2	1	1	1
Enokizono et al. (2022)	2	1	1	3	1	1	2
Greenhill et al. (2001)	3	2	3	3	2	2	3
Gualtieri et al. (1986)	3	1	3	2	3	3	3
Hadar et al. (2021)	2	1	1	1	1	3	2
Humphries et al. (1979)	2	1	1	1	3	3	3
Klass et al. (2012)	2	1	1	2	2	3	2
Leitner et al. (2007)	2	1	1	3	1	3	3
Tucha and Lange (2004)	3	1	3	3	2	3	3
Wade (1976)	2	1	1	2	2	3	2
Werry et al. (1980)	2	1	1	3	2	3	3

Scoring was completed using the Quality Assessment Tool for Quantitative Studies (Thomas et al., 2004): Strong (1), Moderate (2), Weak (3).

### Outcome measures

The studies included in this systematic review employed various outcome measures. A significant barrier to this work is the scarcity of literature using consistent motor measures. Therefore, the lack of standardization of tasks and measures makes direct comparison challenging. Due to the diversity of motor measures and heterogeneity of participants included in each study, a meta-analysis of the data was not feasible. Therefore, this paper presents a summary of the findings in a descriptive manner, along with tabulated summaries. Outcome data from the included studies are summarized in Table 2.

**Table 2:** Studies on the effect of methylphenidate on motor outcomes.

Reference	Methods		Outcomes
	Methylphenidate (dosage, timing)	Comparison (placebo, other)	Motor outcomes
Aitken et al. (2024)	10 mg MPH	Placebo, capsule matching MPH in appearance	The experiments were conducted on a compact driving simulator coupled with UC-win/Road software. There was a difference between the placebo and MPH condition in standard deviation of lateral position across all time points (*p* < 0.01), at thirty minutes (*p* < 0.05), and at 40 min (*p* < 0.01). There was a difference between the placebo and MPH condition in standard deviation of speed at 40 min (*p* < 0.05). There was a difference between the placebo and MPH group in steering variability across all time points (*p* < 0.05).
Barkley (1977)	Hyperkinetic children were given 10 mg MPH and evaluated within 3 h of ingestion. The hyperkinetic and control children were evaluated on three separate visits, with each being separated by a 7- to 14-day interval. Following a baseline (off-MPH) visit, the hyperkinetic children participated in a drug by placebo crossover design in which half received MPH on the second visit and the placebo on the third visit while the other half of the children received MPH and placebo in the reverse order of administration. The hyperkinetic children were randomly assigned to these orders of drug by placebo administration. Although the children discontinued their regular dosage at least 1 day prior to each of the three visits (off-MPH, placebo, and on-MPH days), they were permitted to return to their regular dosage between testing sessions.	The hyperkinetic children were given a placebo and were evaluated within 3 h of ingestion. The control group did not receive any drug or placebo.	Tapping movements were measured via an electric finger tapper device that consisted of a counter affixed to a small wooden base. The counter was activated by depressing a small metal arm. Participants were to tap the arm as rapidly as possible during a 10-s interval using only their index finger while keeping the palm of the hand flat on the wooden base. The number of taps did not differ between MPH and placebo. The maze performance test composed of a half inch raised metal maze, which was connected to an electric timer, counter, and a metal stylus. Participants moved the stylus through the maze as quickly as possible without touching the sides. The apparatus scored the number of contacts made with the sides and the time spent in contact with the sides, while the total time for the trial was manually acquired with a hand-held stopwatch. For hyperkinetic boys, MPH improved the number of contacts (*p* < 0.05) and time in contact with sides during maze coordination test (*p* < 0.01).
Barkley et al. (2005)	Two doses of MPH were tested: a low dose of 10 mg and a high dose of 20 mg. Participants were tested at intervals of 7–14 days. MPH was given to the participant and the participant remained in the lab for 75 min before testing began.	Participants completed a baseline visit, then completed three randomized drug visits: placebo, low dose of MPH, and high dose of MPH. The hospital pharmacy prepared the placebo (lactose powder) and MPH by placing them in orange opaque gelatin capsules to disguise differences in taste between placebo and medication as well as the differences in dose amounts to be employed. The three capsules prepared for each participant were placed in a paper packet corresponding to each day’s medication and numbered to indicate the testing session at which they were to be used with the participant.	The driving simulations were conducted with a virtual reality driving simulator manufactured as a police training simulator. Significant differences were found for the number of crashes, steering variability, course driving time, and the number of turn signals activated. No differences were observed for average driving speed and variability of driving speed. Pair-wise comparisons for crash scores revealed that all three drug conditions resulted in significantly lower crash occurrences than at baseline (*p* < 0.001) but there were no differences between the drug and placebo conditions. For steering variability, variability was greater during the placebo than the baseline condition. In addition, variability was lower during the high dose of MPH than during the placebo condition. No other comparisons were significant. Course driving time was shorter during all three drug conditions relative to baseline (*p* = 0.001); however, no differences were reported between the placebo and drug conditions. For the turn signal score, all three drug conditions resulted in greater use of the turn signal indicator than in the baseline condition (*p* = 0.001). Specifically, the low dose of MPH resulted in significantly greater turn signal usage than in the placebo condition. No additional other pair-wise comparisons for turn signal score were significant. The omnibus test for average driving speed during this course was significant. Pair-wise comparisons indicated that speed during the low dose MPH condition was significantly greater than at baseline, and speed during the placebo condition was greater than at baseline (*p* = 0.074). Average speed during the high dose MPH condition was slower than the low dose MPH condition, and slower than the placebo condition (*p* = 0.069).
Enokizono et al. (2022)	MPH doses ranged from 18 to 54 mg/day (mean dose [SD]: 27.8 mg/day [10.2 mg/day]). The two sessions of data collection were conducted 1–52 days apart (mean interval [SD]: 10.3 days [14.2 days]).	The participants did not take MPH for at least 24 h before the session without MPH.	Finger-tapping tasks were measured using a UB-1, a device that measures the speed and amplitude of finger tapping. The tasks included: in-phase tapping (bimanual, simultaneous tapping) for 10 s, and anti-phase tapping (bimanual alternative) for 10 s. MPH resulted in a lower standard deviation of phase difference in the anti-phase tapping task (*p* = 0.020).
Greenhill et al. (2001)	MPH was administered twice daily, at 08:00 and 12:00 h, and dosage was increased to a ceiling of 60 mg/day or until moderate side-effects developed. This resulted in a mean daily total intake of 39 mg, using twice daily administration. Dosage was adjusted monthly by the physician using teacher reports and scores on the Abbreviated Conners’ Rating Scale. The group began the study with a mean dose of 0.99 mg/kilogram of body weight per day, and it rose to 1.3 mg/kilogram of body weight at 6 months. Participants took 0.9 mg/kilogram of body weight dose at 8 a.m. The protocol schedule yielded four samples for the first hour, three the second hour and one sample per hour for the remaining 7 h, for a total of 14 samples.	A 2-week washout period without MPH, followed by baseline data collection.	A 3-min quantitative measure of motor impersistence and activity level was collected with the Gardner Steadiness Tester (GST). MPH resulted in lower touch times compared to pre-MPH performance (*p* < 0.05). At the 6-month follow up test, the comparison of pre-drug steadiness test and best performance on drug also improved (*p* < 0.01).
Gualtieri et al. (1986)	All participants completed an exercise program involving an exercycle for two 6-day periods (Monday–Saturday), separated by a 1-day washout period (Sunday). One of the conditions was designated ‘intense’ and one ‘light’ exercise. Order of treatments was randomly assigned across subjects. Six participants received intense exercise first and 4 received light exercise first. During each 6-day condition, participants received MPH, 0.2 mg/kilogram of body weight, on 3 days and identical placebo on 3 days.	Placebo matching MPH in appearance.	Participants completed the Purdue Pegboard, a test of manual dexterity, and finger tapping tasks before and after the drug and exercise intervention. For the Purdue Pegboard, the phase by drug interaction was significant (*p* < 0.01), reflecting improved post-test performance in the MPH compared to placebo condition. Similarly, the phase by exercise interaction was significant, (*p* < 0.001) For finger tapping, both the phase by drug, (*p* < 0.01) and the phase by exercise, (*p* < 0.01), interactions were significant.
Hadar et al. (2021)	Assignment to immediate release MPH or placebo was done using a double-blind crossover design. A dosage of 0.3 mg/kilogram of body weight was used. Following 7 days from treatment onset, participants completed a second testing session, which was followed by a 44-h washout period and treatment switch. A week later, the third testing session occurred.	Placebo.	All participants completed the visuomotor attention test at baseline, the control group completed it again at another session, and the study group completed it again during both a placebo and MPH visit. For children with ADHD, difference between MPH and placebo were reported for the ratio between cumulative time the cursor was out of the target and the total test time (*p* < 0.001), mean lateral deviation from the target’s path (*p* = 0.05), number of interruptions (*p* < 0.001), and distance (*p* < 0.005).
Humphries et al. (1979)	An average dose of 12.5 mg MPH was given approximately 1 h before testing. Participants completed morning and afternoon data collections, resulting in a 4-h washout period.	An average dose of 12.5 mg placebo approximately 1 h before testing.	Participants completed two different maze variations, one easy and one difficult, on an Etch-A-Sketch toy. The Etch-A-Sketch consists of a plastic frame enclosing an 18 by 13 cm rectangular screen, below which are two control dials. MPH resulted in less errors compared to placebo (*p* < 0.001), but no differences for time were observed.
Klass et al. (2012)	Participants were randomly assigned to one of three drug treatments. In the MPH condition, participants ingested a placebo (lactose, 20 mg) on the night before the experiment and 40 mg of MPH on arrival in the laboratory.	In the placebo condition, a 20 mg of lactose was ingested, both the night before and the day of the experimental. In the reboxetine condition, participants ingested 8 mg of reboxetine the night before the experiment and 8 mg of reboxetine on arrival in the laboratory.	Participants completed a time trial, a cycling race where each rider races alone against the clock over a set distance. No differences were observed between MPH and placebo conditions for completion time or mean power output. Agonist and antagonist muscle activations for the rectus femoris, vastus medialis, and the biceps femoris were similar prior to exercise for all drug conditions. After exercise, the muscle activation of the agonist muscles decreased for the placebo and reboxetine conditions (*p* < 0.05), but not for the MPH condition. No differences were found in the motor-evoked potential area or the motor-evoked potential latency of the rectus femoris and vastus medialis after exercise for the MPH, reboxetine, or placebo. Measures of voluntary activation, assessed via transcranial magnetic stimulation and motor nerve stimulation, did not differ between the MPH and placebo.
Leitner et al. (2007)	During one data collection, participants took their routine dose of MPH 2 h before they participated in the study. Dosing was optimized for each participant, typically, 5–10 mg of short-acting MPH. Participants were not treated with any other medication except MPH during the study period.	During one data collection, participants were off their prescribed MPH for at least 72 h. During another data collection, participants received a placebo, identical in appearance to MPH, 2 h before they participated in the study.	MPH did not have a significant effect on the average stride time during usual walking or during dual tasking. MPH tended to increase gait speed both during usual walking and during dual tasking (*p* = 0.08). While both on and off MPH, there was a significant reduction in gait speed during dual tasking, compared to the single task condition. Compared to baseline (off MPH), MPH treatment significantly reduced single task walking stride time variability. In contrast to the off-MPH state, after taking MPH, dual tasking no longer significantly affected stride time variability. MPH reduced stride time variability (*p* = 0.001) such that the dual task did not affect variability.
Tucha and Lange (2004)	In Study One, MPH was taken as prescribed, mean total dose was 23.75 mg/day, SEM = 2.27 mg. In Study Two, MPH was taken as prescribed. For children, mean total dose was 22.00 mg/day, SEM = 2.00 mg. For adults, mean total dose was 23.00 mg/day, SEM = 2.26 mg.	Both Study One and Two used a 12-h withdrawal from prescribed dose of MPH. The time between testing sessions was 1 week for children with ADHD and 23 weeks for adults with ADHD.	In Study One, participants wrote the phrase “Ein helles grelles Licht” (“A bright and glaring light”) in cursive script both on their prescribed? stimulant medication and following withdrawal of MPH under three different conditions: normal writing, writing with eyes closed, and writing faster than normal. The position of the pen on the digital tablet, pen velocity, and pen acceleration were measured continuously during writing. MPH resulted in reduced fluency of normal handwriting as indicated by an increased inversions of the direction of the velocity (*p* = 0.012) compared to placebo. Additionally, differences were found between normal handwriting on MPH for both writing with closed eyes (*p* = 0.011) and writing faster than normal (*p* = 0.012). Study Two: Participants were asked to write the same phrase as in the first experiment both on their prescribed MPH and following the withdrawal of MPH. The phrase was written five times per participant. For children with ADHD, MPH resulted in more inversions in the direction of their velocity profiles compared to children without ADHD (*p* = 0.007). Children with ADHD, when not on MPH, did not differ from children without ADHD in the mean number of inversions of the direction of the velocity (*p* = 0.233). MPH resulted in an increased dysfluency during handwriting, as indicated by a higher mean number of inversions of the direction of the velocity (*p* = 0.005) compared to placebo.
Wade (1976)	Dosages ranged from 0.09 mg per kilogram of body weight to 1.17 mg per kilogram of body weight. The dosage levels had been adjusted for each child to a level considered optimum by the research pediatrician. Doses were administered one and a half hours prior to participation in the experiment.	Hyperactive participants completed a 3-week before the placebo data collection session. The control group did not receive MPH or placebo.	Participants balanced in the lateral plane on a 3- by 3-foot board which rotated about a central steel shaft. The goal was to balance for as much of a 30-s trial as possible. For the “time in balance” scores, all the hyperactive subjects, both on MPH and placebo, improved over the two sessions as did the matched control group (*p* < 0.01). Average performance was higher for the hyperactive participants while on MPH than on placebo, irrespective of session. The matched control group performed at a higher level than the hyperactive subjects. The hyperactive subjects on MPH were more consistent in their performance across the two sessions than when on placebo (*p* < 0.01). The performance variability of the matched control group was essentially the same as that of the hyperactive subjects while on MPH.
Werry et al. (1980)	Each child was examined four times: pre-test, MPH, placebo, and imipramine. MPH was given in standardized doses of 0.40 mg/kilogram of body weight for 3–4 weeks.	Details were not provided on the placebo. Imipramine was initially given in doses of 1.00 mg/kilogram of body weight, but after half the children had completed the trial, the dose was increased to 2.00 mg/kilogram of body weight.	Participants complete a maze test and a Graduated Holes Test both adopted from a Motor Steadiness Battery. During the Maze test, the child was required to move a stylus through a maze while attempting to avoid touching the sides. Dependent variables included the number of contacts (i.e., errors) and the total time in contact with the sides (i.e., error time) for both the dominant and non-dominant hands. The graduated holes test required the child to hold the stylus in the center of a series of four holes, graduated in difficulty, without touching the sides. Steadiness was sampled for 10 s on each of the holes, and the child was given a 10-s rest between trials. Dependent variables were the number of contacts and time of contact for the dominant and nondominant hands. The maze task tended to be insensitive to drug effects, although MPH did reduce error time (*p* < 0.05). The Graduated Holes Test was responsive to drug effects with MPH causing reductions in errors (*p* < 0.01) and error time (*p* < 0.05).

ADHD, attention deficit hyperactivity disorder; kg, kilograms; MPH, methylphenidate; mg, milligram; SEM, standard error of the mean.

## Results

### Description of included studies

The 13 articles that met the inclusion criteria and are included in this review comprise five Level 1 RCTs, three Level II nonrandomized studies with two groups, and five Level III one-group nonrandomized studies. Table 2 reports the levels of evidence and the characteristics of the samples for all 13 studies. Of these 13 studies, nine investigated youth cohorts (Wade, 1976; Humphries et al., 1979; Greenhill et al., 2001; Enokizono et al., 2022; Hadar et al., 2021; Werry et al., 1980; Barkley, 1977; Leitner et al., 2007), four investigated adult cohorts (Aitken et al., 2024; Barkley et al., 2005; Klass et al., 2012; Gualtieri et al., 1986), and one study (Tucha and Lange, 2004) included both youth and adult cohorts. A total of 396 individuals participated in the 13 studies, including 267 children and 129 adults. The majority of participants across all included studies were boys or men (*n* = 295, 75%). Six studies included girls or women participants (*n* = 101, 26%), but no statistical comparisons were made between the sexes (Aitken et al., 2024; Tucha and Lange, 2004; Barkley et al., 2005; Werry et al., 1980; Leitner et al., 2007; Humphries et al., 1979). One study did not report the sex of its participants (Wade, 1976). In terms of diagnosis, 10 out of the 13 studies included participants that were diagnosed with hyperactivity, ADD, or ADHD (Wade, 1976; Humphries et al., 1979; Leitner et al., 2007; Tucha and Lange, 2004; Enokizono et al., 2022; Hadar et al., 2021; Werry et al., 1980; Barkley, 1977; Barkley et al., 2005; Greenhill et al., 2002). Five of those 10 studies included a non-hyperactive or non-ADD/ADHD control group (Wade, 1976; Leitner et al., 2007; Tucha and Lange, 2004; Hadar et al., 2021; Barkley, 1977).

As reported in Table 2, administration procedures for MPH varied across articles. In two studies, participants continued to take their routine dose of MPH whilst participating in the research (Leitner et al., 2007; Tucha and Lange, 2004). Four studies administered identical dosages for all participants receiving MPH (Aitken et al., 2024; Klass et al., 2012; Barkley, 1977; Barkley et al., 2005). Five studies prescribed dosage based on participant mass (Wade, 1976; Hadar et al., 2021; Werry et al., 1980; Gualtieri et al., 1986; Greenhill et al., 2001). Two studies reported the average dose received (Humphries et al., 1979; Enokizono et al., 2022). Humphries et al. (1979) described the dosage as “a clinically effective dose of methylphenidate (average dose 12.5 mg)”. The lowest standardized doses administered were 10 mg (Barkley, 1977; Barkley et al., 2005) and 0.0–0.4 mg/kg (Werry et al., 1980). The highest standardized doses administered were 40 mg (Klass et al., 2012) and 0.9 mg/kg (Greenhill et al., 2001).

The timing of MPH administration and the details of MPH washout periods were not consistently reported in the studies reviewed. In the six studies that reported the time between MPH administration and motor testing, some reported a lag of 1 to 3 h between ingestion and motor testing (Wade, 1976; Humphries et al., 1979; Leitner et al., 2007; Barkley, 1977; Barkley et al., 2005). Eight studies reported a washout period prior to placebo testing (Aitken et al., 2024; Hadar et al., 2021; Barkley, 1977; Wade, 1976; Enokizono et al., 2022; Leitner et al., 2007; Humphries et al., 1979; Tucha and Lange, 2004). Two studies reported a washout period of 1 week (Aitken et al., 2024; Hadar et al., 2021). One study reported a washout period of 7–14 days (Barkley, 1977), one reported a 3-week washout (Wade, 1976), one reported a 2-week washout (Greenhill et al., 2001), another reported a 3-day washout (Leitner et al., 2007), and one study reported a 24-h washout (Enokizono et al., 2022). Two studies reported less than a 24-h washout: one with a 4-h washout (Humphries et al., 1979), and another with a 12-h washout (Tucha and Lange, 2004). Below, we report the findings of the 13 studies organized into subgroups based on the type of motor task conducted: gross motor control, fine motor control, and maze tasks.

### Gross motor function

Five of the 13 articles included measures of gross motor function (Aitken et al., 2024; Barkley et al., 2005; Klass et al., 2012; Leitner et al., 2007; Wade, 1976). Three of the five studies used a different gross motor task, thus limiting the ability to compare across studies. These tasks included standing balance (Wade, 1976), gait (Leitner et al., 2007), cycling and muscle contractions (Klass et al., 2012; Leitner et al., 2007; Wade, 1976). Both Aitken et al. (2024) and Barkley et al. (2005) assessed simulated driving.

Wade (1976) compared hyperactive children on MPH and placebo and non-hyperactive children in a standing balance task. In this task, participants were to maintain a stable position on a horizontal balance board for as much of a 30-s trial as possible. Hyperactive participants spent significantly more time in a stable position when on MPH than when on the placebo (Wade, 1976). Age and weight-matched controls spent significantly more time in a stable position compared to hyperactive participants, regardless of the drug condition (Wade, 1976). Hyperactive participants had less variability across the 30 trials in the MPH condition than in the placebo condition, and their variability neared that of matched non-hyperactive controls, but these results were not significant (Wade, 1976). Thus, there is moderate evidence (Level II) to suggest that MPH influences performance on the standing balance task for hyperactive children (Wade, 1976).

Aitken et al. (2024) and Barkley et al. (2005) assessed the effects of MPH on driving performance using simulated driving software and a stationary vehicle base with a seat, dashboard, and steering wheel. Barkley et al. (2005) investigated the influence of a low dose of MPH (10 mg), a high dose of MPH (20 mg), and a placebo on driving performance in two simulated scenarios, a standard course and an obstacle course. The standard course involved following verbal directions and responding to critical events, like a car swerving without warning, while driving through highway, country, and city environments in both day and night conditions (Barkley et al., 2005). The obstacle course involved participants driving as fast as they could down a straight road while avoiding cars placed periodically on the road (Barkley et al., 2005). When on the placebo, participants exhibited greater steering variability in standard driving courses than on the high dosage of MPH. Further, they used turn signals more frequently during both low and high MPH conditions (Barkley et al., 2005). Average speed, speed variability, course driving time, and the number of crashes did not differ between the placebo, low MPH dosage, and high MPH dosage for the standard driving course (Barkley et al., 2005). In the obstacle course, participants had greater average speed when on a low dose of MPH (10 mg) than on a high dose (20 mg); however, speed variability, steering variability, and course driving time did not differ between MPH conditions (Barkley et al., 2005).

Aitken et al. (2024) investigated the influence of a 10 mg dose of MPH and a placebo in adults without ADHD in a standard simulated driving environment, a 105-km, bi-directional, four-lane highway with standard local road markings and signage. Participants weaved less in the MPH condition, indicated by a significantly reduced standard deviation of lateral position compared to the placebo conditions (Aitken et al., 2024). At 40 min of simulated driving, the MPH condition resulted in significantly improved speed maintenance, as indicated by the standard deviation of speed; however, this did not result in a significant difference for the overall simulated driving session (Aitken et al., 2024). Finally, during the MPH condition, there was a reduction in steering wheel movements compared to the placebo condition (Aitken et al., 2024). Overall, there is strong evidence (Level I, Level I) suggesting that MPH influences simulating driving performance for adults with (Barkley et al., 2005) and without ADHD (Aitken et al., 2024).

Leitner et al. (2007) compared gait in children with and without ADHD in single- and dual-task walking. The dual-task walking condition required passive listening for a key word (Leitner et al., 2007). MPH reduced stride time variability for the single-task walking condition (Leitner et al., 2007). In addition, a trend was observed such that MPH resulted in increased gait speed for both the single- and dual-task walking conditions (Leitner et al., 2007). Overall, there is moderate evidence (Level II) to suggest that MPH influences single- and dual-task walking for children with ADHD (Leitner et al., 2007).

Klass et al. (2012) investigated the effects of MPH, reboxetine, and a placebo on cycling performance and neuromuscular function before and after an acute cycling session in a sample of well-trained male cyclist or triathletes without ADHD. No statistically significant differences were observed between MPH and placebo conditions in time trial, a type of cycling race where each rider races alone against the clock over a set distance, completion time or mean power output (Klass et al., 2012). Power output remained stable throughout the time trial in both placebo and MPH conditions (Klass et al., 2012). The agonist and antagonist muscle activations for the rectus femoris, vastus medialis, and the biceps femoris were similar prior to exercise for all drug conditions (Klass et al., 2012). After exercise, the muscle activation of the agonist muscles decreased significantly for the placebo and reboxetine conditions, but not for the MPH condition (Klass et al., 2012). No significant differences were found in the motor-evoked potential area or the motor-evoked potential latency of the rectus femoris and vastus medialis after exercise for the MPH, reboxetine, and a placebo condition (Klass et al., 2012). Measures of voluntary activation, assessed via transcranial magnetic stimulation and motor nerve stimulation, also did not differ significantly between the MPH and placebo conditions (Klass et al., 2012). Overall, there is strong evidence (Level I) to support that MPH does not significantly alter cycling performance or neuromuscular function compared to placebo for well-trained male cyclists or triathletes (Klass et al., 2012).

### Fine motor function

Seven of the 13 studies included measures of fine motor control (Greenhill et al., 2001; Gualtieri et al., 1986; Tucha and Lange, 2004; Werry et al., 1980; Enokizono et al., 2022; Hadar et al., 2021; Barkley, 1977). Fine motor tasks included finger tapping (Barkley, 1977; Gualtieri et al., 1986; Enokizono et al., 2022), placing tests (Gualtieri et al., 1986; Werry et al., 1980), steadiness tests (Greenhill et al., 2001), and handwriting (Tucha and Lange, 2004).

Three studies examined the effects of MPH on finger tapping (Barkley, 1977; Gualtieri et al., 1986; Enokizono et al., 2022). Barkley (1977) investigated the influence of 10 mg of MPH compared to placebo on 10-s intervals of finger tapping for hyperkinetic boys. The mean tapping speeds of both the dominant and non-dominant hand did not differ across the drug conditions (Barkley, 1977). In contrast, Gualtieri et al. (1986) reported that MPH increased the rate of finger tapping compared to placebo for men without ADHD (Gualtieri et al., 1986). Enokizono et al. (2022) compared an MPH condition to a washout condition for boys with ADHD on in-phase and anti-phase tapping. They reported that there was a significant effect of MPH on standard deviation of phase of difference in the anti-phase tapping task, indicating more consistent timing between taps (Enokizono et al., 2022). No other significant differences were reported (Enokizono et al., 2022). Overall, there is moderate evidence (Level II) suggesting that a 10 mg MPH dose administered 3 h before testing does not influence finger tapping for hyperkinetic boys (Barkley, 1977). However, there is also moderate evidence (Level III; Level III) suggesting that MPH does influence finger tapping for children given doses ranging from 18 to 54 mg (Enokizono et al., 2022) and adults without ADHD given a dose based on weight (Gualtieri et al., 1986).

Two studies examined motor performance on manual placing tasks (Gualtieri et al., 1986; Werry et al., 1980). Gualtieri et al. (1986) examined performance of men without ADHD on the Purdue pegboard test (Tiffin and Asher, 1948), a measure of manual dexterity used to assess how effectively participants can manipulate small objects with their hands. Gualtieri et al. (1986) reported that MPH significantly improved Purdue Pegboard performance, both independently and in combination with exercise, as shown by higher post-test scores compared to placebo (Gualtieri et al., 1986). Similarly, Werry et al. (1980) examined hyperactive children’s performance on the graduated holes task, which requires participants to hold a stylus steady in the center of holes with increasing difficulty (i.e., smaller diameters) without touching the sides. Werry et al. (1980) reported the number of errors and error times (i.e., total time in contact with the sides), decreased when participants were on MPH compared to when they were on the placebo. Overall, there is moderate evidence (Level III, Level III) to suggest that MPH improves performance on placing tasks for children with inattention and hyperactivity (Werry et al., 1980) and adults without ADHD (Gualtieri et al., 1986).

Greenhill et al. (2001) evaluated performance on the Gardner Steadiness Test, which measures manual steadiness and fine motor control by requiring participants to hold a stylus within a series of progressively smaller holes, without touching the sides (Greenhill et al., 2001). Greenhill reported reduced touch times, interpreted as improved performance, in the Gardner Steadiness Test when participants were on MPH compared to placebo (Greenhill et al., 2001). Overall, there is moderate evidence (Level III) that MPH improves steadiness in fine motor tasks for boys with ADHD (Greenhill et al., 2001; Tucha and Lange, 2004).

Tucha and Lange (2004) required children with ADHD to write a phrase in six conditions: normal writing, writing with eyes closed, and faster than normal (with eyes open), on MPH, and off MPH. Phase one of this study included eight children with ADHD. In Phase one, MPH reduced fluency of the normal condition; however, during the eyes closed and the faster condition, the writing was more fluent and automated compared to the placebo condition (Tucha and Lange, 2004). Phase two of this study included 10 children and 10 adults with ADHD, as well as 10 children and 10 adults without ADHD (Tucha and Lange, 2004). In Phase two, participants wrote a phrase as they would normally, with their eyes open and at their typical speed, on and off MPH (Tucha and Lange, 2004). For children with ADHD in the off-MPH condition, handwriting did not differ from that of children without ADHD (Tucha and Lange, 2004). However, MPH resulted in significantly more inversions in the direction of velocity profiles for children with ADHD compared to children without ADHD (Tucha and Lange, 2004). Additionally, based on the mean number of inversions of the direction of the velocity, for children with ADHD, MPH resulted in more dysfluent writing compared to the off-MPH condition (Tucha and Lange, 2004). No differences were reported for adults with or without ADHD, on or off MPH (Tucha and Lange, 2004). Overall, there is strong evidence (Level 1) to suggest that MPH may impair handwriting fluency in children with ADHD during typical writing tasks; however, MPH did not have a significant impact on handwriting for adults with ADHD (Tucha and Lange, 2004).

Hadar et al. (2021) investigated the effects of MPH and placebo on the visuomotor attention test (VMAT) for boys with ADHD. During this task, participants use manipulandum to track a continuously moving target and these movements were tracked in two-dimensional coordinates (Hill et al., 2016). Hadar et al. (2021) reported a significant difference for relative time spent out of target, number of interruptions, and the mean distance between the target center and the cursor for the MPH compared to the placebo trials (Hadar et al., 2021). Together, these results demonstrate that MPH resulted in improved performance on the VMAT (Hadar et al., 2021). Overall, there is strong evidence (Level 1) suggesting MPH influences the VMAT for boys with ADHD (Hadar et al., 2021).

### Maze tasks

Three studies assessed maze tracking tasks (Humphries et al., 1979; Werry et al., 1980; Barkley, 1977). Barkley (1977) and Werry et al. (1980) implemented similar maze tasks in which the participant used a stylus to trace through a metal maze. However, Humphries et al. (1979) implemented maze tasks in which participants traced through a maze track using an Etch-A-Sketch^®^.

In Barkley (1977) and Werry et al. (1980), hyperkinetic children and hyperactive boys, respectively, were instructed to use a stylus to trace through a metal maze. The number of contacts made between the stylus and maze walls (i.e., errors) and the time of contact (i.e., error time) were collected for the participants’ dominant and non-dominant hands (Barkley, 1977; Werry et al., 1980). Werry et al. (1980) combined dominant and non-dominant hand performance for analysis, although Barkley (1977) did not. Participants in both studies completed one trial per hand (Barkley, 1977; Werry et al., 1980). Barkley (1977) found that the maze performance of the dominant hand did not differ across placebo and MPH condition (10 mg of Ritalin) in adolescent hyperkinetic males. However, maze performance differed between placebo and MPH conditions for the non-dominant hand such that more errors were made with the non-dominant hand when on the placebo than when on MPH (Barkley, 1977). Time in contact with the sides of the maze was lower in the placebo condition compared to the MPH condition for the non-dominant hand (Barkley, 1977). The total time to complete the maze did not differ between conditions for either hand (Barkley, 1977). Werry et al. (1980) averaged maze performance for the dominant and non-dominant hands and found error time was significantly less in the MPH condition (0.40 mg/kg) compared to the placebo condition for hyperactive children (Werry et al., 1980). However, the number of errors and the completion time was not significantly different for these conditions (Werry et al., 1980).

Humphries et al. (1979) implemented a complex maze task in which participants traced through a maze track using an Etch-A-Sketch^®^ with two dials, one controlling horizontal movement and the other controlling vertical movement (Humphries et al., 1979). Hyperactive children completed six segments on an easy maze and six segments on a complex maze, under each medication condition, placebo and MPH (average dose of 12.5 mg) (Humphries et al., 1979). A significant drug effect was found on the number of errors: participants made significantly less errors during the MPH trials, but there was no significant difference in total time to completion for both the easy and complex maze conditions (Humphries et al., 1979). Overall, there is moderate evidence (Level III, Level III, Level III) suggesting that MPH influences maze performance for hyperactive children (Humphries et al., 1979), children with a prolonged history of inattention and hyperactivity (Werry et al., 1980) and hyperkinetic boys (Barkley, 1977).

## Discussion

The results of this systematic review provide evidence to suggest that MPH positively influences the standing balance task (Wade, 1976), simulated driving performance (Aitken et al., 2024; Barkley et al., 2005), single- and dual-task walking (Leitner et al., 2007), manual placing tasks (Gualtieri et al., 1986; Werry et al., 1980), manual steadiness tasks (Greenhill et al., 2001), the visuomotor attention test (Hadar et al., 2021), and manual maze tasks (Barkley, 1977; Humphries et al., 1979; Werry et al., 1980). In contrast, however, MPH impaired handwriting fluency for children with ADHD during typical writing tasks (Tucha and Lange, 2004). Additionally, MPH did not influence handwriting fluency for adults during typical writing tasks, nor did it influence cycling performance or neuromuscular function for well-trained male athletes (Klass et al., 2012). Finally, the influence of MPH on finger tapping speed is equivocal: Enokizono et al. (2022) and Gualtieri et al. (1986) demonstrated an effect of MPH on consistency (Enokizono et al., 2022) and rate of tapping (Gualtieri et al., 1986), whereas Barkley (1977) reported no effect of MPH on tapping.

Five of the 13 articles included gross motor outcomes: dynamic balance (Wade, 1976), single- and dual-task walking (Leitner et al., 2007), cycling, muscle contractions (Klass et al., 2012), and simulated driving (Aitken et al., 2024; Barkley et al., 2005). Wade (1976) reported that, compared to the placebo, hyperactive children spent more time in balance and had less score variability when on MPH during the standing balance task. These results may be attributed to MPH’s influence on cerebellar processing, improving the processing of sensory inputs (Bucci et al., 2016). Leitner et al. (2007) report that stride-time variability decreased with MPH, and a trend demonstrated that gait speed increased for both single- and dual-task walking with MPH. Importantly, these whole-body tasks involve the basal ganglia and cerebellum, both which increase in neural activation with a single dose of MPH (Czerniak et al., 2013).

Klass et al. (2012) reported that for well-trained athletes, MPH did not significantly alter cycling performance or neuromuscular function compared to the placebo. They hypothesized that MPH would have a significant effect on fatigue, as it would inhibit dopamine reuptake and thereby improve performance (Klass et al., 2012). Although the results did not support this hypothesis, previous work suggests that these improvements might be temperature dependent (Nybo, 2010; Roelands et al., 2008). Therefore, the effects of MPH on neuromuscular function may be, in part, modulated to ambient temperature.

Aitken et al. (2024) and Barkley et al. (2005) reported that MPH positively influenced simulated driving. Specifically, Barkley et al. (2005) reported that the high-dose (20 mg) condition reduced steering variability compared to the placebo condition, and both the low-dose MPH (10 mg) and high-dose (20 mg) conditions resulted in an increased the number of turn signals used compared to the placebo condition during a standard simulated driving course. Additionally, during the obstacle course driving condition, participants had a greater average speed in the low-dose MPH (10 mg) condition compared to the high-dose (20 mg) condition (Barkley, 1977). Similarly, Aitken et al. (2024) report improved speed maintenance, reduced weaving, and a decrease in steering wheel movements during the MPH condition compared to the placebo condition in a standard simulated driving environment. Importantly, driving is a multimodal task, and these performance improvements may be related to MPH’s beneficial effects on oculomotor control (Allman et al., 2012); selective attention (Czerniak et al., 2013; Roberts et al., 2020), and inhibitory control (Czerniak et al., 2013; Roberts et al., 2020).

Six of the 13 studies included fine motor control outcomes: finger tapping (Barkley, 1977; Gualtieri et al., 1986; Enokizono et al., 2022), placing tests (Gualtieri et al., 1986; Werry et al., 1980), steadiness tests (Greenhill et al., 2001), and handwriting (Tucha and Lange, 2004). There are mixed reports on the influence of MPH on finger tapping (Barkley, 1977; Gualtieri et al., 1986; Enokizono et al., 2022). Specifically, Barkley (1977) reported no changes in finger tapping following MPH. However, Enokizono et al. (2022) and Gualtieri et al. (1986) reported differences in finger tapping for children given doses ranging from 18 to 54 mg compared to a washout condition, and for adults without ADHD given a weight-based dose compared to a placebo condition, respectively. The decrease in the standard deviation during antiphase tapping for the MPH condition reported by Enokizono et al. (2022) and the increase in total number of finger taps reported by Gualtieri et al. (1986) may be related to MPH’s ability to enhance response preparation processes by influencing the excitability of the motor cortex (Berger et al., 2018).

Gualtieri et al. (1986) reported that MPH improved Purdue Pegboard scores compared to the placebo condition. Werry et al. (1980) reported improved performance on the graduated holes task for children with a history of inattention and hyperactivity. Specifically, MPH resulted in reduced errors and a shorter time to complete the task compared to the placebo condition (Werry et al., 1980). Similarly, Greenhill et al. (2001) reported reduced touch times in the Gardner Steadiness Test, a task during which participants are asked to hold a stylus within nine holes of diminishing size. Last, Hadar et al. (2021) reported differences between MPH and placebo trials in terms of time spent out of target, number of interruptions, and distance. Importantly, these placing tasks require fine control of fingertip forces, the integration of multi-sensory feedback, and inhibitory control. Although it is well known that MPH enhances inhibitory control in people with (Pietrzak et al., 2006) and without ADHD (Roberts et al., 2020), the specific impact of MPH on force control and sensory-motor integration is unknown.

Three of the 13 studies assessed maze tracking tasks that were, overall, positively affected by MPH (Barkley, 1977; Humphries et al., 1979; Werry et al., 1980). Barkley (1977) reported a decrease in error time and number of errors for the non-dominant hand compared to the placebo in stylus maze tracking. However, total time to complete the maze did not differ between conditions, for either hand (Barkley, 1977). Werry et al. (1980) also examined stylus maze tracking and reported that error time was decreased in the MPH condition compared to the placebo condition. Finally, Humphries et al. (1979) investigated an Etch-A-Sketch^®^ maze task and reported that MPH influenced the number of errors made, but not the total time to complete the task (Humphries et al., 1979). Importantly, maze tasks require sustained attention which is enhanced by MPH (Roberts et al., 2020) and fine motor control, which, as we have shown here, is also generally enhanced by MPH.

Tucha and Lange (2004) investigated handwriting for children and adults with ADHD. For children with ADHD, MPH reduced fluency of the normal writing condition; however, during the eyes closed and the faster condition, the writing was more fluent and automated compared to the placebo condition (Tucha and Lange, 2004). However, no differences were observed for adults with ADHD; and therefore, Tucha and Lange (2004) suggested that MPH does not directly affect writing fluency. An important consideration is task difficulty; specifically, if children’s baseline performance is poor, there is room for improvement. If, on the other hand, adults’ baseline performance is not poor, there may not be room for improvement when MPH is administered. Therefore, the equivocal findings on handwriting necessitate further investigation in standardized studies, especially those that can provide precise kinematic measurements to tease out potential subtle differences in the motor control of handwriting.

Across included studies, the most consistent MPH-related motor improvements were observed in measures of motor variability or steadiness including steering variability (Aitken et al., 2024; Barkley et al., 2005), stride time variability (Leitner et al., 2007), steadiness/error time (Greenhill et al., 2001; Werry et al., 1980; Wade, 1976), bimanual phase variability (Enokizono et al., 2022); however, mean-performance metrics were often unchanged (Barkley et al., 2005; Leitner et al., 2007; Greenhill et al., 2001; Werry et al., 1980; Enokizono et al., 2022). In studies using weight-based moderate dosing schedules, improvements were primarily observed in outcomes related to motor consistency or error reduction (Gualtieri et al., 1986; Hadar et al., 2021; Wade, 1976). Only one study directly compared dose levels (10 vs. 20 mg) (Barkley et al., 2005), suggesting outcome-specific dose sensitivity rather than a uniform dose–response relationship. In other words, MPH effects were most consistently observed for variability measures rather than uniform task improvement. Notably, however, the handwriting studies (Tucha and Lange, 2004) demonstrate that handwriting kinematics reveal mixed or counterproductive effects. Taken together, these findings suggest non-uniform dose effects across motor outcomes. Future studies examining dose–response relationships should carefully consider the task demands and outcome selection in study design.

## Study strengths and limitations

To our knowledge, this is the first systematic review focused on the effects of MPH on quantitative motor outcomes, filling an important gap in the literature. A strength of this systematic review is its rigorous and transparent methodology, including clearly defined inclusion and exclusion criteria and a comprehensive search strategy, which ensures that the synthesis of evidence is as unbiased and thorough as possible.

The available literature on this topic is limited, particularly when applying our inclusion and exclusion criteria, but also highly heterogeneous. Methodological and reporting inconsistencies were common, including variations observed in MPH dosage and timing of administration, differences in washout periods or lack of reporting washout timing, diverse placebo control methods, and substantial variability in outcome measures and tools used. Additional limitations include small sample sizes, the fact that only two of the 13 studies included adults with ADHD, and a lack of sex-based analysis and representation. The considerable variation across existing research introduces substantial challenges for synthesizing findings and limits the ability to draw definitive conclusions about the effects of MPH on motor control.

## Clinical implications

MPH confers its most reliable motor benefits in domains related to variability, steadiness, and error regulation, rather than uniformly improving speed, strength, or overall motor performance. Therefore, MPH may be beneficial for patients that experience inconsistent motor performance or difficulty maintaining stable performance, especially in tasks requiring sustained attention, such as driving. In contrast, we found little evidence that MPH enhances gross motor output or speed-based performance. Notably, the handwriting studies (Tucha and Lange, 2004) indicate that fine motor fluency may be adversely affected by MPH, suggesting that complementary interventions such as occupational therapy may be more appropriate for handwriting tasks. Taken together, these findings suggest that MPH may be beneficial for people with ADHD when motor performance is characterized by inconsistency, excessive variability, or poor error regulation. These findings underscore the task- and outcome-specific nature of MPH’s effect on the motor system.

## Conclusion

This systematic review highlights significant gaps and methodological inconsistencies in the current literature on MPH and motor control. Overall, the findings of this systematic review indicate that MPH generally enhances gross and fine motor performance on tasks requiring attention, response control, motor coordination, and visuomotor integration, though its effects vary by task demands and sample characteristics. Further, the precise mechanism by which MPH enhances motor control is not yet understood and may be related to the inherent cognitive nature of motor tasks. Considerable variation in motor tasks, outcome measures, and dosing protocols introduces challenges for synthesizing findings and limits the ability to draw definitive conclusions. Future studies should implement consistent and clearly reported MPH dosing protocols, including timing of administration and washout periods. Researchers should prioritize the use of standardized and validated motor tasks to facilitate meaningful comparisons across studies. There is also a need for investigations that focus on adults with ADHD and include sex-based analyses to understand potential sex differences in MPH’s effects on motor output.

## Data Availability

The original contributions presented in the study are included in the article/supplementary material, further inquiries can be directed to the corresponding author.

## Footnotes

1
https://www.covidence.org/

## Appendix

Pubmed

(((“Methylphenidate”[Mesh] OR methylphenidate OR aptensio OR Concerta OR Metadate OR Methylin OR Quillivant OR Ritalin OR Ritaline OR Daytrana OR centedrin OR equasym OR phenidylate OR tsentedrin))) AND ((“Psychomotor Performance”[Mesh] OR “Motor Skills”[Mesh] OR “Motor Activity”[Mesh] OR gait[tiab] OR walking[tiab] OR locomot*[tiab] OR (motor[tiab] AND (coordination*[tiab] OR performance[tiab] OR activit*[tiab] OR skill*[tiab]))))

APA PsycINFO

(Methylphenidate OR aptensio OR Concerta OR Metadate OR Methylin OR Quillivant OR Ritalin OR Ritaline OR Daytrana OR centedrin OR equasym OR phenidylate OR tsentedrin) AND (Psychomotor Performance OR motor coordination* OR motor performance OR motor activit* OR motor skill* OR gait OR walking OR locomot*)

International pharmaceutical abstracts

(Methylphenidate OR aptensio OR Concerta OR Metadate OR Methylin OR Quillivant OR Ritalin OR Ritaline OR Daytrana OR centedrin OR equasym OR phenidylate OR tsentedrin) AND (Psychomotor Performance OR motor coordination* OR motor performance OR motor activit* OR motor skill* OR gait OR walking OR locomot*)

ProQuest dissertations and theses

(Methylphenidate OR aptensio OR Concerta OR Metadate OR Methylin OR Quillivant OR Ritalin OR Ritaline OR Daytrana OR centedrin OR equasym OR phenidylate OR tsentedrin) AND (Psychomotor Performance OR motor coordination* OR motor performance OR motor activit* OR motor skill* OR gait OR walking OR locomot*)
